# Attitudes towards mental health and the integration of mental health services into primary health care: a cross-sectional survey among health-care workers in Lvea Em District, Cambodia

**DOI:** 10.1080/16549716.2017.1331579

**Published:** 2017-06-15

**Authors:** Maria Alfredsson, Miguel San Sebastian, Bhoomikumar Jeghannathan

**Affiliations:** ^a^ Epidemiology and Global Health, Department of Public Health and Clinical Medicine, Umeå University, Umeå, Sweden; ^b^ Centre for Child and Adolescent Mental Health, Chey Chumenas Hospital Takhmau, Kandal Province, Cambodia

**Keywords:** Mental health, primary health care, health-care workers, Cambodia, integration

## Abstract

**Background**: Cambodia is a country where the resources for treating mental health disorders are far from sufficient. One strategy to narrow the treatment gap is to integrate mental health into primary health care (PHC). Understanding the knowledge and attitudes towards mental health integration that health-care workers have is important for assessing the challenges and opportunities when planning a potential integration project.

**Objective**: The aim of this study was to assess these basic conditions in Lvea Em District, Cambodia.

**Design**: A structured self-reporting questionnaire regarding attitudes and knowledge about mental health and its integration into PHC was collected from 75 health-care workers in Lvea Em District, Cambodia in October 2015. Firstly, descriptive analyses were carried out, and secondly, linear regression analyses to assess the relationship between attitudes and socio-demographic variables were conducted.

**Results**: There was clear support towards integrating mental health services into PHC among these participants as 81.3% were interested in personally delivering mental health care at their units. Respondents who reported having received some kind of mental health-care training tended to have a more positive attitude towards mentally ill people (*p* = 0.005) and those who thought there was a high need for mental health care had a more favourable attitude towards the integration of mental health services (*p* = 0.007).

**Conclusions**: The most important finding from this survey was the willingness and the acceptance of the need for integration of mental health care. This enhances the feasibility of integrating mental health services at the PHC level. Improving the competence of mental health care in these settings will likely help to reduce the treatment gap for mental, neurological and substance use disorders in Cambodia.

## Background

Mental, neurological and substance use (MNS) disorders are a global problem, affecting 450 million people [1]. As many as 80% of people suffering from MNS disorders are living in low- and middle-income countries (LMICs) [2] and the treatment gap in these settings can be as large as 75–90% [3,4]. This means that a large majority of the people with these disorders are not receiving adequate or any care at all [5]. One strategy to narrow the treatment gap for MNS disorders is to integrate them into primary health care (PHC). The World Health Organization (WHO) has for many years strongly recommended this as the most effective way of making the treatment accessible for patients in settings where specialist mental health workers are scarce [6]. The process of redistributing mental health services from mental health professionals to non-specialist health workers in PHC is called task shifting. The task shifting process is gaining growing importance as its evidence base develops, showing it seems to improve clinical outcomes for common MNS disorders [5,7–10]. At PHC level the preconditions exist for the holistic perspective, for long-lasting health-care continuity [4,11,12], for encompassing geographical coverage and for the possibility to deliver preventive interventions, as well as early detection and treatment for patients with MNS disorders [4,6,8,13,14].

Cambodia is an LMIC in East Asia, with a population of 15 million people. The country has a history of war and conflicts and became very poor after gaining independence from French colonial rule in 1953. The Vietnam War spilled over into Cambodia and the country collapsed during the regime of the Khmer Rouge in the mid-1970s. Millions of people died due to starvation, sickness and executions during the four years of Khmer Rouge rule. In the last two decades Cambodia has seen rapid economic development [15], but still as many as 20.5% of the population is below the poverty line, making Cambodia one of the poorest countries in the region [16].

Social and economic conditions of poverty are linked to common mental illnesses [17]. In post-conflict areas, such as Cambodia, the mental health services have been trauma-focused for a long time, but today the challenge is to include other common mental illnesses in the health-care system [18]. There are only a few population-based prevalence studies on MNS disorders in the country, but there are indications of a high prevalence for many of the common MNS diagnoses [19], together with a high incidence of suicides [20].

Few people are adequately trained for treating mental disorders in the country [21], with around 50 trained psychiatrists [20] and only 1 trained child and adolescent psychiatrist. There are 18 psychiatric beds for the entire country, of which 12 are in private hospitals [21]. In 2010, mental health services were available in only 9 out of 24 provinces [20]. In the same year, 60% of the referral hospitals and 2% of the health centres were able to provide mental health services [22]. Added to the lack of human resources, there are funding constraints as approximately only a meagre 0.02% of the total health budget is assigned to mental health care [23]. In summary, the resources for treating mental disorders in Cambodia are limited, highlighting the huge treatment gap and service need.

The Ministry of Health has recently set up goals to reduce the burden of non-communicable diseases, in which mental health is included. Since the coverage of mental health services is a long way from being sufficient [22,24,25], interventions at the level of PHC will be essential for scaling up these services. However, one of the barriers to the development of mental health services in Cambodia is the complexity of this integration [26], given the lack of resources and adequately trained staff outside the specialized care system.

Primary health-care workers will be key actors in an integration process like this. Understanding their knowledge and attitudes regarding mental illness and mental health-care integration is important for assessing the challenges and opportunities for this potential integration. Stigma is correlated to a decreased treatment effect, a higher probability of relapses [27,28], lack of human resources willing to specialize within the field, and in addition, it creates a barrier to good care service as well as integration [29]. The aim of this study was therefore to assess these basic conditions regarding stigma and attitudes among health workers in Lvea Em District, Cambodia.

## Method

### Setting

**Figure 1.**
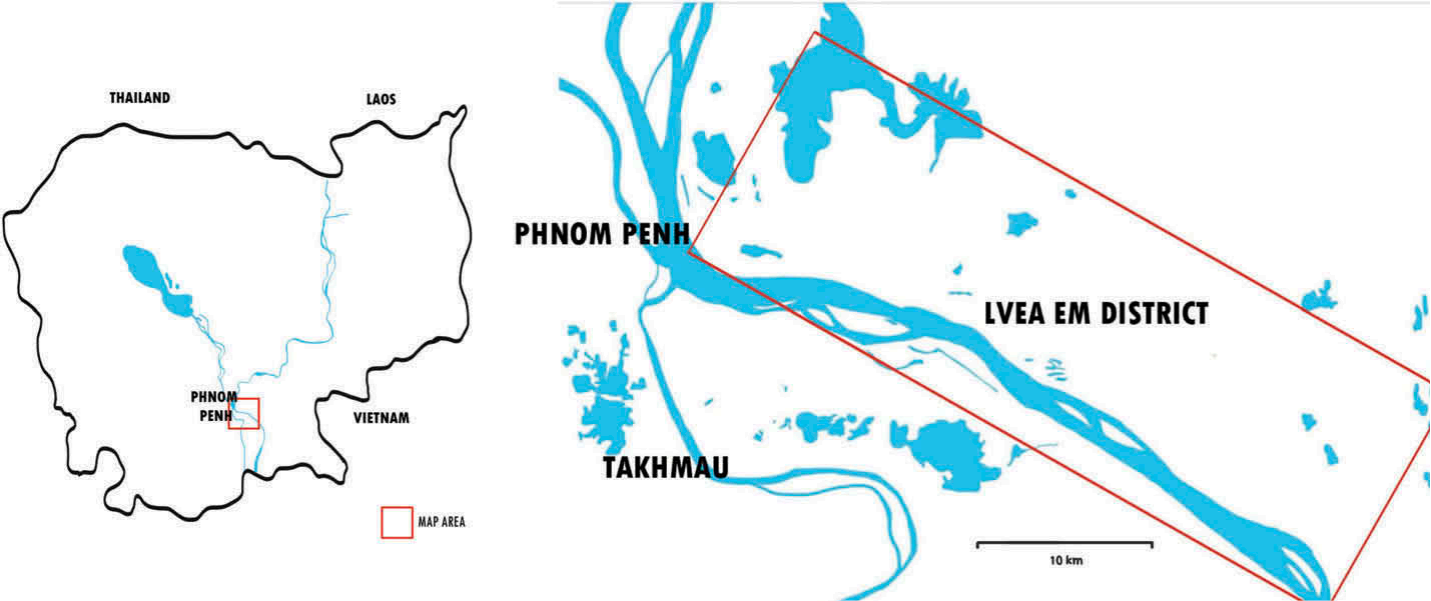
Map of Cambodia and Lvea Em District.

This study took place at the 11 health centres and the district hospital in Lvea Em District, Kandal Province, Cambodia (Figure 1). The district is a rural area located on the north riverbed of the Mekong, which is accessible by ferry from the southern part of Phnom Penh. The district has a population of approximately 86,000, living in 43 villages, predominately making their living from farming [30]. There is no mental health service provided in the district today [25]. The district was chosen since there was already a plan for an integration process to take place here. While the district represents the settings and resources of the rural area in Cambodia, the relatively closer location to the capital, where some resources for mental health care can be found, makes it particular.

The participants included in this study were employees of the health-care system at any of the health-care facilities in the district. In total there were 80 health-care workers in the district (information provided by the district health officer), supported by community health-care workers. Mainly midwives and nurses are stationed at the PHC centres, generally providing maternal care and child health care. Medical doctors are available at the district referral hospital, where basic treatment for common disorders like diarrhoea and acute respiratory infections is provided. On the other side of the Mekong River, the provincial referral hospital in Takhmau provides specialized care. Also located in Takhmau is Cambodia’s only referral centre for child and adolescent mental health and related problems, the Centre for Child and Adolescent Mental Health (CCAMH). The closest referral hospital for adult mental health problems is situated in Phnom Penh, 10 km north of Takhmau, where the country’s only psychiatric inpatient unit is located [20].

The study was performed in cooperation with the staff of CCAMH. The centre has been operational since 1991 as a collaborative project between the Ministry of Health of Cambodia and Caritas Cambodia, an international non-governmental organization (NGO).

### Instrument

A self-reporting questionnaire was developed, based on instruments used in previous studies for attitudes about mental illnesses. Socio-demographic characteristics were investigated regarding education, training and the health-care workers’ experience of the mental health-care situation in their district. Attitudes towards mental illness and its integration into PHC were analysed using statements used in previous researches [31,32]. The statements regarded the participants’ opinions about people with mental illnesses and attitudes concerning working with mental health care. The participants were asked to state whether they agreed with, disagreed with or were neutral about the statements.

The questionnaire was designed with mainly optional answers and just a few open questions. The mental health professionals working at CCAMH, who were native Khmer speakers, helped to translate it into Khmer. The questionnaire was first pretested among five health-care workers in the district and during discussion with them several changes were made. This was to adapt the questionnaire to fit the local context and to ensure that the formulation of the questions was adapted to the education level of the health-care workers.

### Data collection

Data collection was carried out in October 2015 by the first author together with mental health professionals working at CCAMH. The data were collected from health-care workers at the 11 health-care centres and the district hospital in Lvea Em. The questionnaire was handed out to 75 health-care workers and all of them were included in the study. The author met 38 of the respondents to administer the questionnaire. The remaining 37 participants received the questionnaires and their instructions through their colleagues.

### Data analysis

Data analysis was performed using the software tool ‘Epi info 7’. Data were analysed first descriptively. The attitude component was analysed by developing two indexes to obtain a summary of the current attitudes. The first index described *stigma and attitudes towards mental illness*, a summary of eight statements, and the second index described *attitude towards integration of mental health into PHC*, including five statements. Each answer to a statement that indicated a positive attitude was coded as 1, while an answer indicating a negative attitude was coded as −1. The values of each statement were then summarized together to create an index value for each participant. A higher value thus indicated a more positive attitude towards mental illness and mental health-care integration. Linear regression analyses were performed to assess the relationship between socio-demographic variables and the attitude indexes. All significant variables (*p* < 0.05) were included in subsequent multivariable linear regression models.

## Results

### Socio-demographic characteristics

Overall information was collected from all 75 health workers. More than two-thirds (76.0%; n = 57) of the participants were female and 75.7% (n = 56) were married. Most of the respondents (97.3%; n = 73) were Buddhists. The mean age was 35.7 years (SD 11.4; median 30.0), and 59.4% (n = 41) had a diploma-level education while 40.6% (n = 28) had a degree-level education. As many as 41.7% of the participants had been working in clinical settings for more than 10 years (Table 1).

**Table 1.** Characteristics of respondents (*n* = 75).

Variables	N (%)
Gender	
Female	57 (76.0)
Male	18 (24.0)
	
Age (years)	
Mean (SD)	35.7 (11.4)
18–29	33 (47.1)
30–39	11 (15.7)
40–49	16 (22.9)
50–59	9 (12.9)
60+	1 (1.4)
	
Marital status	
Single	16 (21.6)
Married	56 (75.7)
Divorced or widowed	2 (2.7)
	
Religion	
Buddhist	73 (97.3)
Christian	2 (2.7)
	
Educational level	
Diploma^a^ Degree^b^	41 (59.4) 28 (40.6)
	
Profession	
Diploma clinical nurse	12 (16.9)
Degree clinical nurse	14 (19.7)
Midwife	36 (50.7)
Medical doctor	2 (2.8)
Other^c^	7 (9.7)
	
Years of clinical experience	
1–2	20 (27.8)
3–5	13 (18.1)
5–10	9 (12.5)
> 10	30 (41.7)
	

Notes: ^a^One-year training program after high school.

^b^Three-year training program after high school.

^c^Students and staff performing various medical tasks including vaccination and distribution of medication without any specific education within the medical field.

### Training and mental health situation

One-third of the respondents (32.0%; n = 24) reported that they had received some kind of training in mental health care. Among these respondents a majority reported that the training was regarding child mental health, through CCAMH-organized workshops. Almost half of the respondents (44.3%; n = 31) declared that they had some knowledge about mental illness from their university studies, but the most frequently reported source for knowledge about mental illness was public media (61.4%, n = 43).

When asked what resources were missing regarding mental health care, 94.5% (n = 69) reported better knowledge and training of the staff. Almost one-third (28.8%; n = 21) thought that medication for mental illness was lacking and should be provided at PHC level. A clear majority of the respondents (90.3%; n = 65) thought that mental health care was needed or very much needed in their area. Around half of the respondents (56.8%; n = 42) declared that they had met adults during the last year that they believed had some kind of mental illness. A lower proportion of the participants, 32.9% (n = 24), had met children with the same problems (Table 2).

**Table 2.** Training and setting for mental health service.

Question	Answer N (%)
Received training in mental health care?	
Yes	24 (32.0)
No	51 (68.0)
	
Resources missing regarding mental health care at your health care center?	
Better knowledge and training of the staff	69 (94.5)
Medication	21 (28.8)
	
Source of knowledge about mental illness?	
University study	31 (44.3)
Public media	43 (61.4)
	
Need of mental health care in your area?	
Very much needed	16 (22.2)
Needed	49 (68.1)
No need	7 (9.7)
	
Have you met adults at this facility that you believe can	
have some kind of mental illness during the last year?	
Yes	42 (56.8)
No	32 (43.2)
	
Have you met children at this facility that you believe can	
have some kind of mental illness during the last year?	
Yes	24 (32.9)
No	49 (67.1)
	

### Stigma and attitudes towards mental illness

Table 3 shows that a large proportion of the respondents thought that mental illness was a problem for Cambodia and there was a consistent belief that mental health care is important. Many of the participants (66.7%; n = 50) agreed with the statement that mentally ill people are dangerous and show unpredictable behaviour. Some 23.3% (n = 17) stated that people with mental illness should not be allowed to work and 33.8% (n = 25) thought that mentally ill people should not be allowed to have children. However, it is encouraging that 68.9% (n = 51) disagreed with the statement ‘Mental health is less important than physical health’.

**Table 3.** Health-care workers’ stigma and attitudes towards mental illness.

Statement	Agree N (%)	Neutral N (%)	Disagree N (%)	Total N
Mental illness is a problem for Cambodia	67 (90.5)	3 (4.1)	4 (5.4)	74
Mental health care is important	73 (98.7)	1 (1.3)	0 (0.0)	74
Traditional healers are better in effectiveness than our medical care	2 (2.7)	4 (5.4)	68 (91.9)	74
Mental illness is not curable and delivering mental health care is wasting resources	3 (4.1)	10 (13.5)	61 (82.4)	74
People with mental illness have unpredictable behavior and are dangerous	50 (66.7)	9 (12.0)	16 (21.3)	75
People with mental illness should not be allowed to work	17 (23.3)	15 (20.5)	41 (56.2)	73
People with mental illness should not be allowed to have children	25 (33.8)	16 (21.6)	33 (44.6)	74
Mental health is less important than physical health	18 (24.3)	5 (6.8)	51 (68.9)	74

**Figure 2.**
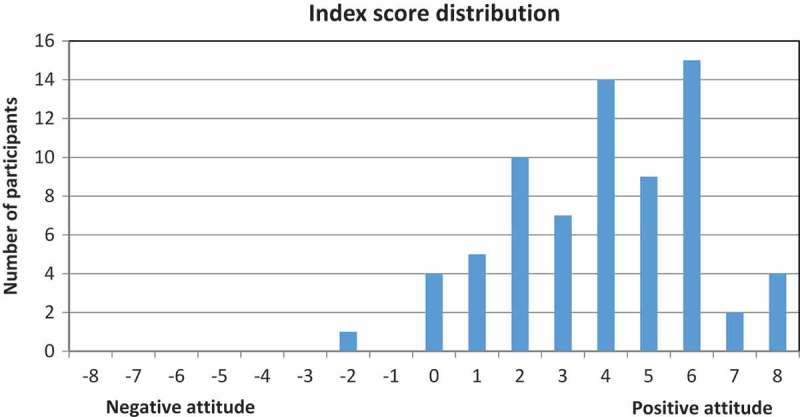
Distribution of index scores for stigma and attitudes towards mental illness.

An index describing a summary of these statements regarding stigma and attitudes towards mental illness was constructed and calculated for 71 participants. Four participants were excluded due to missing answers in one or more of the index statements. Figure 2 illustrates the index score distribution among the responders. The responders’ index score was between −2 and +8, with the distribution of the scores towards the more positive part of the axis. The mean value for this index was +4.0 (median 4.0; SD 2.2). Only one (1.4%) participant had a negative value when summarizing the answers.

Four factors were significantly associated with a more positive attitude and less stigma in the univariate analysis (Table 4): (1) the opinion that there is a need for mental health services in the district; (2) having received some training about mental health care; (3) profession, i.e. the medical doctors – the group of medical doctors, however, only comprised two participants; and (4) having seen adults that might have problems with mental illness. There was, however, no such association among those respondents that had reported seeing children with the same kinds of problems. In the multivariable analysis the only significant associations found for a more positive attitude were being a medical doctor and having received some training about mental health care (Table 4).

**Table 4.** Association between attitudes towards mental illness and socio-demographic variables.

	Univariate analysis		Multivariate analysis	
Variable	β coefficient	*p*-value	β coefficient	*p*-value
Age	−0.029	0.233		
Sex (ref ‘Female’) Male	0.906	0.142		
Years of clinical experience	−0.217	0.318		
Marital status (ref ‘Single’) Married	−0.100	0.876		
Educational level (ref ‘Degree’) Diploma	−0.752	0.178		
Profession (ref ‘Degree clinical nurse’)				
Diploma clinical nurse	0.250	0.766		
Medical doctor^1^	3.833	0.017*	4.388	0.006**
Midwife	–0.252	0.714		
Have you, during the last year, met adults at this health care center that you believe can have some kind of mental illness? (ref ‘No’) Yes	1.392	0.008**	0.529	0.366
Have you, during the last year, met children at this health care center that you believe can have some kind of mental illness? (ref ‘No’) Yes	0.130	0.820		
Received any training in mental health care (ref ‘No’) Yes	1.386	0.012*	1.684	0.005**
Self-estimated need of mental health				
service in the area (ref ‘No need’)				
Needed	2.312	0.016*	1.186	0.254
Very much needed	3.000	0.005**	0.570	0.633

*p < 0.05, **p < 0.01.

^1^This group only included two participants.

### Attitudes towards integration of mental health services into PHC

As many as 77.0% of the participants thought that health-care centres can play an important role in improving the mental health service, 78.7% were interested in having mental health services integrated into their facility and 81.3% showed interest in personally delivering mental health care (Table 5).

**Table 5.** Health-care workers’ attitudes towards integration of mental health services into primary health care.

Statement	Agree N (%)	Neutral N (%)	Disagree N (%)	Total N
Health care centers can play an important role in improving the mental health care in Cambodia	57 (77.0)	11 (14.9)	6 (8.1)	74
Would you be interested to have mental health care integrated into your health center?	59 (78.7)	8 (10.7)	8 (10.7)	75
Are you personally interested in actually delivering mental health care in your health center?	61 (81.3)	10 (13.3)	4 (5.3)	75
Delivering mental health service in the health centers will put other patients to danger	9 (12.3)	9 (12.3)	55 (75.3)	73
Mentally ill patients should not be treated in the same health center with the general patients	31 (41.3)	10 (13.3)	34 (45.3)	75

**Figure 3.**
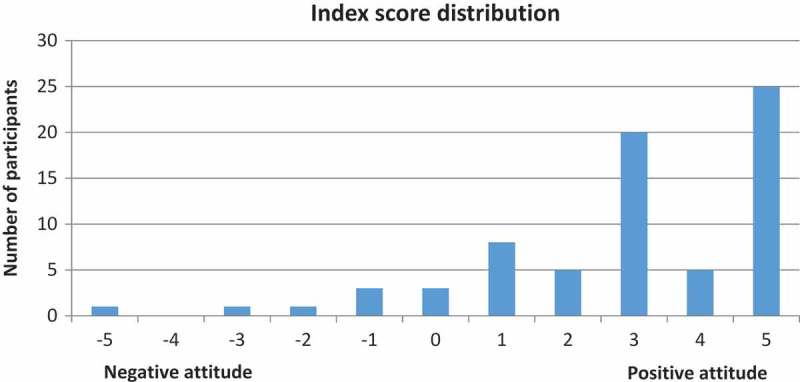
Distribution of index scores for attitudes towards integration of mental health service into primary health care.

In total, 72 participants were included in constructing the index describing the overall attitude towards the integration of mental health services into PHC. Three participants were excluded due to missing answers. Like the previous index, the integration index was distributed towards the more positive part of the axis (Figure 3). The responder’s index score was distributed between −5 and +5. The mean value for this index was +2.9 (median 3.0; SD 2.2). A proportion of 8.3% (n = 6) of the participants scored a negative value.

Similarly to the previous score for stigma, an association with a more positive attitude towards integration was found in the univariate analysis for those health-care workers who estimated a higher need for mental health services in the villages, as well as for those who reported having met people with mental health problems (Table 6). For this index there was also an association with age. In the multivariable analysis, only considering a higher need for mental health services remained associated with the integration index.

**Table 6.** Association between attitudes towards integration of mental health care into primary health care and socio-demographic variables.

Variable	β coefficient	*p*-value	β coefficient	*p*-value
	Univariate analysis		Multivariate analysis	
Age	0.053	0.026*	0.027	0.257
Sex (ref ‘Female’)				
Male	0.778	0.194		
Years of clinical experience	0.074	0.728		
Marital status (ref ‘Single’)				
Married	0.625	0.322		
Educational level (ref ‘Degree’)				
Diploma	−0.722	0.197		
Profession (ref ‘Degree clinical nurse’)				
Diploma clinical nurse	−0.214	0.795		
Medical doctor^1^	1.786	0.263		
Midwife	–0.214	0.747		
Have you, during the last year, met adults at this health care center that you believe can have some kind of mental illness? (ref ‘No’)				
Yes	1.306	0.012*	0.723	0.179
Have you, during the last year, met children at this health care center that you believe can have some kind of mental illness? (ref ‘No’)				
Yes	0.966	0.079		
Received any training in mental health care (ref ‘No’)				
Yes	0.211	0.713		
Self-estimated need of mental health				
service in the area (ref ‘No need’)				
Needed	2.362	0.029*	1.860	0.084
Very much needed	4.000	0.001**	3.318	0.007**

*p < 0.05, **p < 0.01.

^1^ This group only included two participants.

## Discussion

This study explored health-care workers’ attitudes towards mental illness and the integration of mental health services into PHC in a rural district in Cambodia. There were some tendencies to stigmatize beliefs among this studied group. However, more importantly, there was a general positive attitude towards, and willingness for, future integration of mental health services into non-specialized facilities.

### Stigma and attitudes towards mental illness

Despite the general positive attitude towards mental illness, 66.7% considered mentally ill people to be unpredictable and dangerous. This is slightly more than in similar studies on attitudes from Zambia [32]. However, the reason why the health-care workers had this opinion was not further investigated in this study. Significant proportions of the respondents agreed with the statements that people with mental illness should not be allowed to work (23.3%) and not be allowed to have children (33.8%). An interesting topic for further studies would be whom the health-care workers have in mind when they refer to ‘people with mental illness’. It is possible that the participants might be referring to people with acute psychosis or mania when they consider them to be potentially more dangerous. These are, however, stigma-related thoughts against MNS disorders and it is important that health-care workers are made aware of their own attitudes and the way they may produce and reproduce them [29,32,33] as well as the effect of negative attitudes on the quality of the care service [29].

A more favourable attitude was found among respondents who had identified mental illness recently in their area and assessed that there was a need for mental health care. A relationship between a positive attitude towards mentally ill people and personally knowing someone suffering from such a disease is well known from the literature [34]. The lack of association found for age, gender and marital status was also similar to previous studies [31].

In this study the only association found between profession and positive attitude was for those with the highest level of education, the medical doctors, similarly to findings in the sub-Saharan region [35,36] and Nepal [37]. This group did however only comprise two participants, which makes the result uncertain. The lack of a stronger association with other levels of education could be explained by the very limited training for mental health within all parts of the educational health system in Cambodia. According to younger graduate medical doctors, psychiatry has recently been included in their education (personal communication). The nursing schools are also supposed to have included theoretical courses for basic training, but the practical value of this education is unlikely to be enough according to the national strategic plan [22]. Greater knowledge and more highly trained health workers with regard to mental health care have previously been associated with more favourable attitudes [31]. Those participants in this study who reported having received some kind of medical training regarding mental health care, regardless of their educational level, did however indicate having less stigma towards mental illness. Continuing the education for PHC workers is probably essential for enhancing their attitude towards, and competence in, taking care of patients with mental illness.

### Attitudes towards integration of mental health services into PHC

The overall willingness for integration of mental health services into primary care is similar to that found by other studies carried out in Zambia and Nepal [38,39]. Since the health-care workers in these settings are the most crucial human resource for scaling up the MNS health care [5], this is an important finding.

In a study from Zambia, the cited reasons why integration would be beneficial were that the patients would be more willing to access care, care would be brought closer to the communities and the human resources for mental health would increase [38]. Logistical challenges where community members have emphasized the money and time to be saved for the patients have been seen in Uganda [9]. Also, in this study the health-care workers commented that it would be better if medication could be provided at the health centres, to enhance the availability of treatment. In the study district, it is necessary to visit a specialized unit in Phnom Penh to receive any mental health service. Every time a patient needs a prescription for mental health-related medication it takes all day, because of the inaccessible infrastructure. Often a family member is accompanied, resulting in the loss of daily income for two people. The health-care workers in Lvea Em District, for example, identified a problem with relapses for psychotic patients who could not afford to go to the city to get their medication. This becomes an economic barrier to receiving mental health care in low-income and rural settings. Providing medicine at PHC level, as suggested by the health-care workers, would enhance the availability of medications for patients and probably improve treatment compliance.

### Training and mental health situation

Reduced stigma and more education alone are not enough to provide mental health care. The health-care workers of Lvea Em District were unanimous in their belief that better knowledge and training of the staff at the health-care centres are essential for scaling up the quality of mental health services. For a successful integration attention needs to be paid to *how* the scale-up process should be achieved and what kind of training is relevant. Intervention tools, like mhGAP (the Mental Health GAP Action Programme), can be used and there is an ongoing process to evaluate its long-term outcomes and ways of making improvements [40]. Evidence shows that the training needs to be relevant to its context with continuing in-service education and effective supervision [7,9,40]. In Cambodia in the mid-1990s 600 nurses and doctors were taking part in a 3-month course on psychiatry primary care, initiated by NGOs, but today only 10% of them remain working within this kind of service [22]. In a future scaling-up project one needs to reconsider how to keep the human resources and knowledge already acquired in place, including awareness and prevention of the risk for health-care workers to become overburdened when one more task is shifted onto them [9].

Adequate guidance and leadership are essential for developing the mental health policy as well as coordinating funding and training [41]. International agencies and NGOs have been very influential in the developing of Human Resources for Health (HRH) in Cambodia, as well as contributing with financing. In the 1990s there were conflicting agendas between the different actors and lack of coordination. However, today a more coordinated approach seems to have emerged [42,43]. There is a Mental Health Strategic Plan where the ambition is to reduce the burden of MNS disorders and where key challenges are highlighted [22]. Several deficiencies are identified, e.g. the lack of drugs used for psychiatric treatment, the shortage of competent staff, the inadequate infrastructure, the lack of screening tools and guidelines and the very limited financial resources, including low salaries for staff working with mental health. A recent overview of the mental health situation in Lvea Em District confirms this situation [25]. Cambodia already has experience from scaling up projects within PHC. In the 2000s the country’s maternal care was facing several similar deficiencies regarding human and financial resources [44], and the approach from this successful work can also be adopted when it comes to development of the mental health care.

All the potential challenges need to be kept in mind. The overall positive attitudes from the health-care workers in this study do however contribute to an optimistic outlook for future implementation programmes and the improvement of mental health care. A governmental commitment to improving human competence and the availability of appropriate medication could make mental health care accessible in the settings where a majority of the Cambodian population lives today, and thus help reduce stigma-related barriers as well.

### Limitations

The questionnaire used in this study was not validated but based on previous studies. By using a validated survey the usability is ensured, analyses become more trustworthy and follow-up more reliable. Another limitation was that there was no back-translation of the questionnaire, which may have caused possible conceptual and semantic errors.

Not all of the participants got the opportunity to meet the researcher along with the translator, which could have made it difficult for them to seek clarification if they had any questions. This may have affected the quality of the answers. However, the participants who received their questionnaires with second-hand instructions were compared with those who got first-hand instructions and no significant differences in attitudes were found between the two groups. Among the second-hand-instructed participants there were however significantly more missing answers than from the first-hand-instructed group.

We are aware that some of the answers could have been influenced by the health workers’ working conditions or the health system’s organization. However, this potential bias could not be assessed since the study did not collect information about the structure and functioning of the district health-care system.

## Conclusion

To the best of our knowledge, this study is the first of its kind in Cambodia, and contributes to understanding the health-care workers’ perspectives on mental health. Certain stigmatizing attitudes towards mentally ill people were found among the health workers. However, the willingness and acceptance of the need for integration were high. This is clearly positive in terms of the feasibility of integrating mental health services into the PHC. A concrete next step could be to develop mental health care training for the health-care workers which, in addition to a government commitment to extend the mental health care infrastructure, would most likely help to reduce the treatment gap for MNS disorders in Cambodia.

## Paper context

One strategy to narrow the treatment gap for mental disorders is to integrate mental health services into primary health care. Health-care workers are instrumental in that integration but their attitudes towards such an integration are unknown in Cambodia. The results of this study indicated positive attitudes and therefore a favourable setting for such an integration project in Lvea Em District. Its implementation will contribute to strengthening the mental health-care services and reducing the treatment gap among mentally ill people.
